# Supplementary material for: Identification of novel avian and mammalian deltaviruses provides new insights into deltavirus evolution
Source: Virus Evol. 2021 Feb 12;7(1):veab003. doi: 10.1093/ve/veab003 (PMC7882216; doi:10.1093/ve/veab003)
Supplement: veab003_Supplementary_Data [file veab003_supplementary_data.zip › Supplementary_Tables.pdf]

Supplementary Table 1. Summary of novel deltaviruses (full version)

Virus name	Host species		Tiussue	SRA accession	DDBJ accession	Contig length (nt)	GC content (%)	BLASTx best hit		
	Species name	Common name						Virus name	Accession	Identity (%)
Taeniopygia guttata DeV	*Taeniopygia guttata*	Zebra finch	Scapulohumeralis caudalis	SRR2545946	BR001665	1706	56.6	Rodent deltavirus	QJD13558	63.3
Marmota monax DeV	*Marmota monax*	Eastern woodchucks	Liver	SRR2136906	BR001661	1712	53.4	Hepatitis delta virus	AIR77039	60.0
Odocoileus virginianus DeV	*Odocoileus virginianus*	White-tailed deer	Pedicle	SRR4256033	BR001662	1690	56.4	Hepatitis delta virus	AHB60712	66.7
Erythrura gouldiae DeV	*Erythrura gouldiae*	Gouldian finch	Skin	SRR7504989	BR001660	596	59.4 [a)]	Rodent deltavirus	QJD13555	63.5
Serinus canaria-associated DeV	*Serinus canaria*	Domestic canary	Skin	SRR2915371	BR001664	761	54.4 [a)]	Hepatitis delta virus	AIR77012	36.0

a) GC content of the partial genome sequences.

Supplementary Table 2. Annotation of novel deltaviruses

Virus name	Position		Annotation	Amino acids	Isoelectric point
	Start	End			
tgDeV	1623	1030	DAg ORF	197	10.35
	969	964	Poly(A) signal		
	689	773	Genomic ribozyme		
	920	833	Antigenomic ribozyme		
mmDeV	1629	1048	DAg ORF	193	10.40
	997	992	Poly(A) signal		
	704	792	Genomic ribozyme		
	946	858	Antigenomic ribozyme		
ovDeV	1607	1023	DAg ORF	194	10.63
	973	968	Poly(A) signal		
	701	783	Genomic ribozyme		
	922	835	Antigenomic ribozyme		
scDeV	756	139	DAg ORF	205	10.63
	90	85	Poly(A) signal		
egDeV	574	<1 [a)]	DAg ORF	>191	10.44

a) The ORF continues to outside of the obtained contig.

Supplementary Table 3. Detection of deltavirus-derived reads in RNA-seq data (full version)

Virus	BioProject/ BioStudy	SRA	Host			RPM (read per million)	Mappped read number	Total read number	Tissue
			Common name	Taxonomy					
				Family	Species				
tgDeV	PRJDB3398	DRR087390	Zebra finch	Estrildidae	*Taeniopygia guttata*	0.02	2	93,969,226	Brain
	PRJEB28085	ERR2772428				0.50	4	8,000,000	Brain
		ERR2772429				0.50	4	8,000,000	Brain
		ERR2772431				0.50	4	8,000,000	Brain
		ERR2772432				0.25	2	8,000,000	Brain
	PRJNA297576	SRR2545941				0.07	4	60,507,624	Pectoralis
		SRR2545942				0.09	6	64,901,474	Pectoralis
		SRR2545943				10.28	541	52,608,066	Pectoralis
		SRR2545944				1.02	70	68,926,452	Scapulohumeralis caudalis
		SRR2545945				0.50	30	60,435,950	Scapulohumeralis caudalis
		SRR2545946				56.73	2,666	46,997,966	Scapulohumeralis caudalis
		SRR2545950				0.07	4	53,458,024	Scapulohumeralis caudalis
	PRJNA352507	SRR5001843				0.02	1	46,262,572	Spleen
		SRR5001847				0.04	2	48,914,708	Spleen
		SRR5001848				0.05	2	37,604,058	Spleen
		SRR5001849				0.04	2	48,240,788	Spleen
		SRR5001850				0.73	42	57,660,048	Spleen
		SRR5001851				0.22	12	53,968,342	Spleen
	PRJNA435424	SRR6761870				0.17	2	12,007,672	Blood
	PRJNA413749	SRR6151592	Black-headed bunting	Emberizidae [a)]	*Emberiza melanocephala*	0.11	2	18,837,970	Liver
	PRJNA558524	SRR9899549	Black-headed bunting		*Emberiza melanocephala*	3.11	81	26,076,780	Blood
	PRJNA470787	SRR7244621	Rufous-fronted bushtit	Aegithalidae	*Aegithalos iouschistos*	0.03	2	78,465,516	Liver
		SRR7244624	Rufous-fronted bushtit			0.03	2	70,294,336	Cardiac muscle
		SRR7244629	Yellow-bellied tit	Paridae	*Pardaliparus venustulus*	0.01	1	72,167,958	Lung
		SRR7244650	Grey crested tit	Paridae	*Lophophanes dichrous*	0.03	2	79,286,956	Liver
		SRR7244673	Rufous-vented tit	Paridae	*Periparus rubidiventris*	0.03	2	62,012,430	Lung

		SRR7244693	Yellow-bellied tit	Paridae	*Pardaliparus venustulus*	10.68	666	62,346,176	Lung
		SRR7244695				2.07	125	60,358,382	Kidney
		SRR7244696				2.65	176	66,475,684	Cardiac muscle
		SRR7244697				7.12	521	73,205,926	Flight muscle
		SRR7244698				1.77	147	82,849,574	Liver
		SRR7244699	Rufous-vented tit		*Periparus rubidiventris*	0.03	2	64,743,790	Cardiac muscle
		SRR7244728	Black-throated bushtit	Aegithalidae	*Aegithalos concinnus*	0.02	1	61,061,634	Cardiac muscle
	PRJNA478907	SRR7504989	Gouldian finch	Estrildidae	*Erythrura gouldiae*	1.07	51	47,450,142	Skin
mmDeV	PRJNA291589	SRR2136864	Woodchuck	Sciuridae	*Marmota monax*	0.04	3	79,395,232	Liver
		SRR2136865				0.06	4	72,004,528	Liver
		SRR2136883				0.07	4	60,179,086	Liver
		SRR2136906				70.86	5,146	72,619,384	Liver
		SRR2136907				63.08	4,358	69,088,520	Liver
		SRR2136908				0.24	11	46,237,666	Liver
		SRR2136909				0.05	2	40,860,302	Liver
		SRR2136910				0.03	2	75,353,994	Liver
		SRR2136911				0.04	2	47,486,488	Liver
		SRR2136912				0.16	8	51,207,898	Liver
		SRR2136913				0.03	2	69,102,274	Liver
		SRR2136916				1.02	57	55,685,944	Liver
		SRR2136917				0.90	38	42,002,154	Liver
		SRR2136918				0.07	4	58,069,234	Liver
		SRR2136981				0.04	2	47,345,042	Liver
		SRR2136982				0.08	8	101,425,488	Liver
		SRR2136998				0.04	2	47,208,236	Liver
		SRR2136999				0.06	2	34,882,902	Liver
	SRP011132	SRR437934				46.34	43	927,972	PBMC
		SRR437938				19.83	22	1,109,424	PBMC
ovDeV	PRJNA317745	SRR4256026	White-tailed deer	Cervidae	*Odocoileus virginianus*	0.16	10	63,121,148	Antler
		SRR4256031				0.18	12	66,438,392	Muscle
		SRR4256033				180.73	8,456	46,787,594	Pedicle
		SRR4256034				0.07	5	74,468,272	Testis
scDeV	PRJNA300534	SRR2915371	Domestic canary	Fringillidae	*Serinus canaria*	9.79	1,723	176,049,404	Skin

a) Emberizidae is regarded as the subfamily Emberizinae of the family Fringillidae in TimeTree.

Supplementary Table 4. Coexisting viruses in deltavirus-positive SRAs (full version)

SRA accession	Host Species	Host Common name	Query contig Name	Length (nt)	Subject Accession	Virus name	Identity	Query Start	Query End	Subject Start	Subject End	E-value	Bitscore	Notes
SRR2545944	*Taeniopygia guttata*	Zebra finch	NODE_79_length_906_cov_4.390832	906	AUN86682	Serinus canaria polyomavirus	100.0	1	735	112	356	6.47E-178	505	
SRR5001849	*Taeniopygia guttata*	Zebra finch	NODE_709_length_1400_cov_37.345427	1400	AUN86682	Serinus canaria polyomavirus	100.0	1349	282	1	356	0	726	
			NODE_754_length_1362_cov_1.354086	1362	AUN86683	Serinus canaria polyomavirus	96.2	1250	3	84	501	0	767	
			NODE_1282_length_1068_cov_3.859738	1068	AUN86680	Serinus canaria polyomavirus	100.0	82	1068	1	329	1.69E-167	481	
SRR5001850	*Taeniopygia guttata*	Zebra finch	NODE_348_length_1959_cov_5.914984	1959	AUN86682	Serinus canaria polyomavirus	95.5	671	1738	1	356	0	677	
SRR5001851	*Taeniopygia guttata*	Zebra finch	NODE_297_length_1923_cov_5.157638	1923	AUN86682	Serinus canaria polyomavirus	100.0	1261	194	1	356	0	726	
SRR2915371	*Serinus canaria*	Common canary	NODE_4_length_5248_cov_65.731580	5248	YP_009041461	Canary bornavirus 3	99.7	5248	125	6	1713	0	3300	
			NODE_157_length_1443_cov_104.502196	1443	YP_009041460	Canary bornavirus 3	98.5	3	1379	43	501	0	909	
			NODE_260_length_1228_cov_1158.091225	1228	YP_009041458	Canary bornavirus 3	100.0	1141	536	1	202	1.89E-127	375	
			NODE_300_length_1177_cov_3498.696364	1177	YP_009041456	Canary bornavirus 3	100.0	41	1153	1	371	0	769	
			NODE_1081_length_795_cov_9.143454	795	NP_573443	Canary circovirus	96.7	705	79	42	250	7.02E-149	426	
			NODE_1298_length_743_cov_2.166667	743	NP_573442	Canary circovirus	97.9	743	36	55	290	2.90E-172	486	
SRR7504989	*Erythrura gouldiae*	Gouldian finch	NODE_114_length_1793_cov_16.827506	1793	YP_009508817	Erythrura gouldiae polyomavirus 1	95.3	1129	44	1	362	0	717	
SRR7244695	*Pardaliparus venustulus*	Yellow-bellied tit	NODE_2107_length_3092_cov_24.285332	3092	ANE10779	Mammalian rubulavirus 5	100	128	1654	1	509	0	1021	Contamination? [a]
			NODE_14943_length_1034_cov_35.282353	1043	YP_009551871	Rhinolophus gammaherpesvirus 1	33.673	704	997	16	111	6.98E-10	68.2	Simple repeat [b]
			NODE_15702_length_996_cov_3.342252	996	AGT76392	Human mastadenovirus C	98.616	49	915	1	289	2.49E-174	494	Contamination? [a]
			NODE_3342_length_3230_cov_7.390929	3230	ANE10783	Mammalian rubulavirus 5	99.819	1670	18	1	551	0	951	Contamination? [a]
			NODE_7583_length_1982_cov_6.047265	1982	ANE10784	Mammalian rubulavirus 5	99.466	210	1895	4	565	0	1043	Contamination? [a]
			NODE_17287_length_870_cov_7.236057	870	YP_009551871	Rhinolophus gammaherpesvirus 1	33.673	555	262	16	111	4.83E-10	67.8	Simple repeat [b]
SRR7244697	*Pardaliparus venustulus*	Yellow-bellied tit	NODE_21247_length_602_cov_5.924453	602	YP_009551871	Rhinolophus gammaherpesvirus 1	33.673	178	471	16	111	1.51E-10	67.8	Simple repeat [b]
SRR7244698	*Pardaliparus venustulus*	Yellow-bellied tit	NODE_8399_length_1418_cov_4.649735	1418	YP_009551871	Rhinolophus gammaherpesvirus 1	33.673	316	609	16	111	1.37E-09	67.8	Simple repeat [b]

a) These sequences showed 98-100% identities to human-derived viruses, and thus are highly probably derived from contamination or index hopping.

b) These sequences consist of direct simple repeats.

Supplementary Table 5. Primers and probes used in this study

Name	Sequence (5'-3')	Note
tgDeV.F1	CTTCGGTGGAATGGACTCTC	Real-time PCR detection of tgDeV from specimens
tgDeV.R1	CAAGCGAAGAAGGCAAGAAG	
tgDeV.PCR.F1	TTGGGACCCTTTTTCTTCGG	End-point PCR detection of tgDeV-like viruses
tgDeV.PCR.R1	GACCCTTTCTCTGAGAGCAC	
tgDeV.PCR.F2	TAACCCGAGAAAAGACCCAATGC	PCR and sequencing of lsDeV
tgDeV.PCR.R2	AGGTGTCTTCAGCAATCCTCTCC	
tgDeV.PCR.F3	CTTATAGGAGCGGCGGGGATAAC	PCR and sequencing of lsDeV
tgDeV.PCR.R3	CTTAAGGGGAGGTTGGAACTCGG	
tgDeV.PCR.F4	AAAGAGCAAGGAACTACGGAGGC	PCR and sequencing of lsDeV
tgDeV.PCR.R4	GCTCTGATCCATTGACAGTTGCC	
tgDeV.PCR.F5	TCGATTAACTTGCCCGTCTGTTG	PCR and sequencing of lsDeV
tgDeV.PCR.R5	TGCTTACCCCGTAGATGTCTGTG	
tgDeV.PCR.F6	CGAGGAGGTTCGAATGTCCGATG	PCR and sequencing of lsDeV
tgDeV.PCR.R6	GCAAGCGAAGAAGGCAAGAAGC	
tgDeV.PCR.F7	TTGGGAGTCGGGAACAAATCCTC	PCR and sequencing of lsDeV
tgDeV.PCR.R7	GAAGGTGGAGACATGGATGTGGA	
tgDeV.PCR.F8	GTCGGGACGATCCTCCATCTCC	PCR and sequencing of lsDeV
tgDeV.PCR.R8	GGCCGACTCTGTCTTCTTTGAGA	
tgDeV.PCR.F9	CGGGTTCGAGGCGAGCTACC	PCR and sequencing of lsDeV
tgDeV.PCR.R9	CCGTGCTCGTCCACAAGGTAAG	
mmDeV.F1	CTTCTTTCTCTAGCCTCATCCG	Real-time PCR detection of mmDeV from specimens
mmDeV.R1	GAACATCCTGGGAATGGTGAG	
tgDeVAg-F	GAAACATTCCCTCCCCACGGGTG	Detection of tgDeV from the cells
tgDeVAg-R	GGACGCAACTCCGAAGAAAAAGGGTC	
mmDeVAg-F	GGAACTCCTGCTTTCCTCTAATG	Detection of mmDeV from the cells
mmDeVAg-R	GGAGAATCCTAAGCAAGGAAAC	
GAPDH-F	CCATGGAGAAGGCTGGGG	Detection of GAPDH from the cells
GAPDH-R	CAAAGTTGTCATGGATGACC	
human HDV-qF	GGACCCCTTCAGCGAACA	
human HDV-qR	CCTAGCATCTCCTCCTATCGCTAT	Quantification of human HDV from the cells
human HDV-qProbe	FAM-AGGCGCTTCGAGCGGTAGGAGTAAGA-TAMRA	
tgDeV-qF	GAAACATTCCCTCCCCACGGGTG	
tgDeV-qR	GACCCTTTTTCTTCGGAGTTGCGTCC	Quantification of tgDeV from the cells
tgDeV-qProbe	FAM- GGACGCAACTCCGAAGAAAAAGGGTC(MGB)	
mmDeV-qF	CTCTTTCTCACCATTCCCAGG	
mmDeV-qR	GGAGACTCTAAGACAATGGGTC	Quantification of mmDeV from the cells
mmDeV-qProbe	FAM- TTCCTCCTCGAGCTCCTCCTTTCT(MGB)	
